# Supplementary material for: UK Patient Access to Low-Protein Prescription Foods in Phenylketonuria (PKU): An Uneasy Path
Source: Nutrients. 2025 Jan 22;17(3):392. doi: 10.3390/nu17030392 (PMC11820046; doi:10.3390/nu17030392)
Supplement: Supplementary file 1 [file nutrients-17-00392-s001.zip › S1 - Patient access to low protein prescription foods in PKU_ a questionnaire for dietetics.pdf]

Patient access to low protein prescription foods in PKU: a questionnaire for dietetics

14 Nov 2024

Hi, would you mind taking 30 minutes to complete this form? We need as many people as possible who are involved in accessing these products for patients to complete this survey. Please answer individually based on your own experience and submit your response by March 21 2024.

* Required

1. Which hospital do you work at? *

2. Are you a: *

- ◯ Dietitian
- ◯ Dietetic Assistant
- ◯ Secretary/Administrator

3. Please state how many days a week that you work *

- ◯ 1 day
- ◯ 2 days
- ◯ 3 days
- ◯ 4 days
- ◯ 5 days

4. Do you look after patients with PKU? *

- ◯ Yes
- ◯ No
- ◯ Yes, no direct contact (secretary/administrator)
- ◯ Yes, no direct contact (research role)

5. Where in the UK do the majority of your PKU patients live (**tick all that apply**)? *

- ☐ London
- ☐ Midlands/Central England
- ☐ North East England
- ☐ North West England
- ☐ Yorkshire and Humber
- ☐ South East England
- ☐ South West England
- ☐ Scotland
- ☐ Wales
- ☐ Northern Ireland
- ☐ Other

6. Are you a dietetic prescriber? *

- ◯ Yes
- ◯ No

7. In your area of the UK are **<u>all patients</u>** (adults and children, irrespective of age or financial circumstance) exempt from prescription charges? *

- ◯ Yes
- ◯ No
- ◯ Unable to answer

8. Do you have any comments regarding question 7?

9. Do you normally care for paediatric or adult patients with PKU, or both? *

- ◯ Paediatric
- ◯ Adult
- ◯ Both

Paediatric Patients

10. Approximately how many **paediatric patients with PKU** does your hospital care for? *

- ◯ 0
- ◯ <10
- ◯ 10-20
- ◯ 21-50
- ◯ 51-100
- ◯ 101-150
- ◯ >150

11. How are LOW PROTEIN PRESCRIPTION FOODS usually prescribed for your **paediatric patients**? *

- ◯ Dietitian prescriber (independent of GP)
- ◯ GP prescription
- ◯ Other

12. Who usually supplies your **paediatric patients** their LOW PROTEIN PRESCRIPTION FOODS? *

- ◯ Chemist/local pharmacy only
- ◯ One home delivery company only
- ◯ Two home delivery companies
- ◯ Three home delivery companies
- ◯ A combination of home delivery companies and chemist
- ◯ Other

13. Do you have any comments about the previous question?

14. How often do you get a message or communication from caregivers of **paediatric patients** to report that they have problems accessing LOW PROTEIN FOOD PRESCRIPTIONS? *

- ◯ At least once a week
- ◯ Once every 2 weeks
- ◯ Once a month
- ◯ Rarely
- ◯ Unaware this is an issue

15. Do you have any comments about the previous question?

16. What are the most common issues caregivers of **paediatric patients** get in contact with you about the supply of LOW PROTEIN FOODS? (**tick all that apply**) *

- ☐ Missing foods
- ☐ Received out of date foods
- ☐ Product is unavailable
- ☐ Not received the amount of food ordered
- ☐ Run out of food supply
- ☐ Not received the correct foods
- ☐ Received too much product
- ☐ Waiting long period before receive low protein foods
- ☐ Received damaged foods
- ☐ GP refused to prescribe some foods
- ☐ Need information about how to re-order foods
- ☐ Other

17. Do you have any comments about the previous question?

18. What percentage of your **paediatric patients** have recurring problems accessing LOW PROTEIN PRESCRIPTION FOODS (eg. inadequate supply, missing products, delayed supply)? *

- None
- ≤5%
- ≤10%
- ≤25%
- ≤50%
- ≤75%
- ≤100%
- Unable to answer

19. Do you have any comments about the previous question?

20. How much time in a typical week <u>do you spend</u> trying to **organise** LOW PROTEIN PRESCRIPTION FOODS for your **paediatric patients**? *

- < 1 hour
- 1-2 hours
- 3-5 hours
- > 5 hours

21. Do you have any comments about the previous question?

22. How much time in a typical week <u>do you spend</u> trying to **correct** LOW PROTEIN PRESCRIPTION FOOD problems for your **paediatric patients**? *

- < 1 hour
- 1-2 hours
- 3-5 hours
- > 5 hours

23. Do you have any comments about the previous question?

24. Which LOW PROTEIN PRESCRIPTION FOOD(S) do your **paediatric patients** most commonly run out of? (**tick all that apply**) *

- [ ] Bread/rolls – fresh & vacuum packed
- [ ] Pasta/Rice
- [ ] Flour/Bread mix
- [ ] Milk replacements
- [ ] Crackers/Biscuits/Cookies
- [ ] Burger/Sausage/Savoury mixes
- [ ] Breakfast cereals
- [ ] Breakfast bars
- [ ] Egg replacer
- [ ] Pancake mix
- [ ] Cake mix
- [ ] Ready meals/Snack pots
- [ ] Pizza bases
- [ ] Crisps/Chocolate

25. Choose the top 5 most common reasons reported by caregivers of **paediatric patients** for running out of LOW PROTEIN PRESCRIPTION FOODS? *

Please select at most 5 options.

- ☐ Issues with the manufacturer supply
- ☐ GP/prescription refusal
- ☐ GP/prescription error
- ☐ GP unable to find low protein product on their computer system
- ☐ Pharmacist unable to find product so they can order it
- ☐ Out-of-date product delivered to pharmacy
- ☐ Prescription sent to wrong place by GP surgery
- ☐ Delivery company delays in delivery
- ☐ Wrong products delivered
- ☐ Misinformation about availability of products
- ☐ Products not available on patient app

26. How often do you hear about a **manufacturers** delay in supply of LOW PROTEIN PRESCRIPTION PRODUCTS for **paediatric patients** in your clinic? *

- ○ At least once a week
- ○ Once every 2 weeks
- ○ Once a month
- ○ Rarely
- ○ Unaware that this is an issue

27. Do you have any comments about the previous question?

28. How often do **GP/prescription** issues usually occur with LOW PROTEIN PRESCRIPTION PRODUCTS for **paediatric patients** in your clinic? *

- ◯ At least once a week
- ◯ Once every 2 weeks
- ◯ Once a month
- ◯ Rarely
- ◯ Unaware that this is an issue

29. Do you have any comments about the previous question?

30. Choose the top 5 most common problems reported by caregivers of **paediatric patients** with **GP practices** when obtaining LOW PROTEIN PRESCRIPTION FOODS? *

Please select at most 5 options.

- ☐ Refusal to prescribe some products requested
- ☐ The number of items requested are reduced
- ☐ Product choice/variety restricted
- ☐ Prescription delays
- ☐ Not able to request products newly available on prescription
- ☐ Errors on prescription - missing items
- ☐ Errors on prescription - incorrect items
- ☐ Errors on prescription - too many items
- ☐ Prescription always on repeat with no flexibility to change it
- ☐ Refusal to do a repeat prescription
- ☐ Prescription not sent to correct supplier
- ☐ GP unable to find low protein product on their computer system

31. Who do you contact in the **GP service** when you have problems with GP prescriptions for LOW PROTEIN FOODS for your **paediatric patients**? *

- ☐ GP
- ☐ Practice manager
- ☐ Practice receptionist
- ☐ Prescription clerk
- ☐ Practice pharmacist
- ☐ Other

32. Do you have any comments about the previous question?

33. How often do you find out that **chemist/pharmacy issues** have occurred with LOW PROTEIN PRESCRIPTION PRODUCTS for your **paediatric patients**? *

- ○ At least once a week
- ○ Once every 2 weeks
- ○ Once a month
- ○ Rarely
- ○ Unaware that this is an issue

34. Do you have any comments about the previous question?

35. Choose the top 5 most common problems reported by caregivers of **paediatric patients** with **chemists/pharmacies** when obtaining LOW PROTEIN PRESCRIPTION FOODS?
*

Please select at most 5 options.

- ☐ Lack of supply from manufacturers/wholesalers
- ☐ Lack of knowledge as to the importance of low protein foods for the patient
- ☐ Accessing prescription from GP
- ☐ Poor communication regarding delivery dates/time
- ☐ Poor communication between GP and chemist
- ☐ Delayed deliveries
- ☐ Missed deliveries
- ☐ Missing items in the delivery
- ☐ Wrong items in delivery
- ☐ Poor communication with caregivers when products not available
- ☐ Prescription not sent to correct supplier
- ☐ Unable to find prescription on NHS spine
- ☐ Damaged packaging
- ☐ Broken or damaged food product
- ☐ Out of date foods
- ☐ Foods close to use-by
- ☐ Unable to source some low protein foods

36. How often do you receive communication from caregivers of **paediatric patients** about **home delivery company issues** with LOW PROTEIN PRESCRIPTION FOODS?
*

- ◯ At least once a week
- ◯ Once every 2 weeks
- ◯ Once a month
- ◯ Rarely
- ◯ Unaware that this is an issue

37. Do you have any comments about the previous question?

38. Choose the top 5 most common problems caregivers of **paediatric patients** report with **home delivery companies** when obtaining LOW PROTEIN PRESCRIPTION FOODS? *

Please select at most 5 options.

- ☐ Lack of supply from manufacturers/wholesalers
- ☐ Accessing prescription from GP
- ☐ Poor communication regarding delivery dates/time
- ☐ Poor communication between GP and home delivery company
- ☐ Delayed deliveries
- ☐ Missed deliveries
- ☐ Missing items in delivery
- ☐ Wrong items in delivery
- ☐ Poor communication with dietitians when products not available
- ☐ Inedible fresh products due to delay in delivery
- ☐ Unable to find prescription on NHS spine
- ☐ Damaged packaging
- ☐ Broken or damaged food product
- ☐ Out of date foods
- ☐ Food close to use-by

39. Which home delivery service do your **paediatric patients** use for their LOW PRESCRIPTION FOODS? (**tick all that apply**) *

- ☐ Vitaflo at Home (Vitaflo and Fate products)
- ☐ Homeward (Nutricia/Loprofin products)
- ☐ Dial a Chemist (Mevalia, Promin, Taranis, Metax products)
- ☐ Not applicable

40. Do you have any comments about the previous question?

41. How often are you informed that a GP has refused to **send a paediatric patient prescription** for LOW PROTEIN FOODS directly to companies? You may have been informed by the GP practice, parent or home delivery company. *

- At least once a week
- Once every 2 weeks
- Once a month
- Rarely
- Unaware that this is an issue

42. Do you have any comments about the previous question?

43. How often **are you** contacted by **home delivery companies** to ask for your help in obtaining a prescription for SPECIAL LOW PROTEIN FOODS for a **paediatric patient** from the GP practices? *

- At least once a week
- Once every 2 weeks
- Once a month
- Rarely
- Unaware that this is an issue

44. Do you have any comments about the previous question?

45. Commonly GP prescriptions are electronically uploaded to the NHS spine (digtal platform) and this may lead to the prescription for LOW PROTEIN FOODS being downloaded by the wrong supplier e.g. incorrect home delivery company or to a local pharmacy instead of a **designated home delivery company**. How often are you aware that this happens with your **paediatric patients**? *

- At least once a week
- Once every 2 weeks
- Once a month
- Rarely
- Unaware that this is an issue

46. Do you have any comments about the previous question?

47. Does your hospital supply LOW PROTEIN FOOD samples to **paediatric patients** if they run out? *

- At least once a week
- Once every 2 weeks
- Once a month
- Rarely
- Unaware that this is an issue

48. Do you have any comments about the previous question?

49. What are the most common issues related to caregivers of **paediatric patients** that affect the supply of LOW PROTEIN PRESCRIPTION FOODS? (**tick all that apply**) *

- ☐ Do not order low protein foods in a timely way
- ☐ Do not collect low protein foods in a timely way
- ☐ Don’t respond to messages from delivery company/chemist
- ☐ Stockpile low protein foods
- ☐ Do not understand ordering process
- ☐ Other

50. Do you have any comments about the previous question?

51. Do **GP practices/local prescribers** place any restrictions on the prescription of LOW PROTEIN FOODS for your **paediatric patients**? *

- ○ Yes
- ○ No

52. If yes, please describe those restrictions. *

53. What would help in the future to make things better for caregivers of **paediatric patients** in accessing LOW PROTEIN PRESCRIPTION FOODS? (**tick all that apply**) *

- ☐ Apps specifically produced for ordering special low protein foods
- ☐ Dietitians prescribing
- ☐ Centralisation of system i.e. one supplier
- ☐ Credit card/voucher system for ordering
- ☐ Improved education of GP surgeries/ practice pharmacist
- ☐ Improved liaison with GP surgery/ pharmacist by dietitian/department
- ☐ Improved education of patient’s local pharmacy
- ☐ Other

54. Do you have any comments about the previous question?

55. What is your perception of the impact of prescription issues on your **paediatric patients** blood phenylalanine control? *

- Never impacts
- Rarely impacts
- Sometime impacts
- Frequently impacts

56. Do you have any comments about the previous question?

57. Do you also care for adult patients with PKU *

- Yes
- No

58. Do you have any final comments?

Adult Patients

59. Approximately how many **adult patients with PKU** does your hospital care for? *

- ◯ <10
- ◯ 10-20
- ◯ 21-50
- ◯ 51-100
- ◯ 101-150
- ◯ >150

60. How are LOW PROTEIN PRESCRIPTION FOODS usually prescribed for your **adult patients**? *

- ◯ Dietitian prescriber (independent of GP)
- ◯ GP prescription
- ◯ Other

61. Who <u>usually</u> supplies your **adult patients** their LOW PROTEIN PRESCRIPTION FOODS? *

- ◯ Chemist/local pharmacy only
- ◯ One home delivery company only
- ◯ Two home delivery companies
- ◯ Three home delivery companies
- ◯ A combination of home delivery companies + chemist
- ◯ Other

62. Do you have any comments about the previous question?

63. How often do you get a message or communication from **adult patients** to report that they have problems accessing LOW PROTEIN FOOD PRESCRIPTIONS? *

- ◯ At least once a week
- ◯ Once every 2 weeks
- ◯ Once a month
- ◯ Rarely
- ◯ Unaware this is an issue

64. Do you have any comments about the previous question?

65. What are the most common issues **adult patients** get in contact with you about the supply of LOW PROTEIN FOODS? (**tick all that apply**) *

- ☐ Missing foods
- ☐ Received out of date foods
- ☐ Product is unavailable
- ☐ Not received the amount of food ordered
- ☐ Run out of food supply
- ☐ Not received the correct foods
- ☐ Received too much product
- ☐ Waiting long period before receive low protein foods
- ☐ Received damaged foods
- ☐ GP refused to prescribe some foods
- ☐ Need information about how to re-order foods
- ☐ Other

66. Do you have any comments about the previous question?

67. What percentage of your **adult patients** have recurring problems accessing LOW PROTEIN PRESCRIPTION FOODS (eg. inadequate supply, missing products, delayed supply)? *

- None
- ≤5%
- ≤10%
- ≤25%
- ≤50%
- ≤75%
- ≤100%
- Unable to answer

68. Do you have any comments about the previous question?

69. How much time in a typical week do you spend trying to **organise** LOW PROTEIN PRESCRIPTION FOODS for your **adult patients**? *

- < 1 hour
- 1-2 hours
- 3-5 hours
- > 5 hours

70. Do you have any comments about the previous question?

71. How much time in a typical week do you spend trying to **correct** LOW PROTEIN PRESCRIPTION FOOD problems for your **adult patients**? *

- < 1 hour
- 1-2 hours
- 3-5 hours
- > 5 hours

72. Do you have any comments about the previous question?

73. Which LOW PROTEIN PRESCRIPTION FOOD(S) do your **adult patients** most commonly run out of? (**tick all that apply**) *

- ☐ Bread/rolls – fresh & vacuum packed
- ☐ Pasta/Rice
- ☐ Flour/Bread mix
- ☐ Milk replacements
- ☐ Crackers/Biscuits/Cookies
- ☐ Burger/Sausage/Savoury mixes
- ☐ Breakfast cereals
- ☐ Breakfast bars
- ☐ Egg replacer
- ☐ Pancake mix
- ☐ Cake mix
- ☐ Ready meals/Snack pots
- ☐ Pizza bases
- ☐ Crisps/Chocolate

74. Choose the top 5 most common reasons reported by **adult patients** for running out of LOW PROTEIN PRESCRIPTION FOODS? *

Please select at most 5 options.

- [ ] Issues with the manufacturer supply
- [ ] GP/prescription refusal
- [ ] GP/prescription error
- [ ] GP unable to find low protein product on their computer system
- [ ] Pharmacist unable to find product so they can order it
- [ ] Out-of-date product delivered to pharmacy
- [ ] Prescription sent to wrong place by GP surgery
- [ ] Delivery company delays in delivery
- [ ] Wrong products delivered
- [ ] Misinformation about availability of products
- [ ] Products not available on patient app

75. How often do you hear about a **manufacturers** delay in supply of LOW PROTEIN PRESCRIPTION PRODUCTS for **adult patients** in your clinic? *

- ○ At least once a week
- ○ Once every 2 weeks
- ○ Once a month
- ○ Rarely
- ○ Unaware that this is an issue

76. Do you have any comments about the previous question?

77. How often do **GP/prescription** issues usually occur with LOW PROTEIN PRESCRIPTION PRODUCTS for **adult patients** in your clinic? *

- ◯ At least once a week
- ◯ Once every 2 weeks
- ◯ Once a month
- ◯ Rarely
- ◯ Unaware that this is an issue

78. Do you have any comments about the previous question?

79. Choose the top 5 most common problems reported by **adult patients** with **GP practices** when obtaining LOW PROTEIN PRESCRIPTION FOODS? *

Please select at most 5 options.

- ☐ Refusal to prescribe some products requested
- ☐ The number of items requested are reduced
- ☐ Product choice/variety restricted
- ☐ Prescription delays
- ☐ Not able to request products newly available on prescription
- ☐ Errors on prescription - missing items
- ☐ Errors on prescription - incorrect items
- ☐ Errors on prescription - too many items
- ☐ Prescription always on repeat with no flexibility to change it
- ☐ Refusal to do a repeat prescription
- ☐ Prescription not sent to correct supplier
- ☐ GP unable to find low protein product on their computer system

80. Who do you contact in the **GP service** when you have problems with GP prescriptions for LOW PROTEIN FOODS for your **adult patients**? *

- ☐ GP
- ☐ Practice manager
- ☐ Practice receptionist
- ☐ Prescription clerk
- ☐ Practice pharmacist
- ☐ Other

81. Do you have any comments about the previous question?

82. How often do you find out that **chemist/pharmacy issues** have occurred with LOW PROTEIN PRESCRIPTION PRODUCTS for your **adult patients**? *

- ○ At least once a week
- ○ Once every 2 weeks
- ○ Once a month
- ○ Rarely
- ○ Unaware that this is an issue

83. Do you have any comments about the previous question?

84. Choose the top 5 most common problems reported by **adult patients** with **chemists/pharmacies** when obtaining LOW PROTEIN PRESCRIPTION FOODS? *

Please select at most 5 options.

- [ ] Lack of supply from manufacturers/wholesalers
- [ ] Lack of knowledge as to the importance of low protein foods for the patient
- [ ] Accessing prescription from GP
- [ ] Poor communication regarding delivery dates/time
- [ ] Poor communication between GP and chemist
- [ ] Delayed deliveries
- [ ] Missed deliveries
- [ ] Missing items in the delivery
- [ ] Wrong items in delivery
- [ ] Poor communication with caregivers when products not available
- [ ] Prescription not sent to correct supplier
- [ ] Unable to find prescription on NHS spine
- [ ] Damaged packaging
- [ ] Broken or damaged food product
- [ ] Out of date foods
- [ ] Foods close to use-by
- [ ] Unable to source some low protein foods

85. How often do you receive communication from **adult patients** about **home delivery company issues** with LOW PROTEIN PRESCRIPTION FOODS? *

- ○ At least once a week
- ○ Once every 2 weeks
- ○ Once a month
- ○ Rarely
- ○ Unaware that this is an issue

86. Do you have any comments about the previous question?

87. Choose the top 5 most common problems **adult patients** report with **home delivery companies** when obtaining LOW PROTEIN PRESCRIPTION FOODS? *

Please select at most 5 options.

- [ ] Lack of supply from manufacturers/wholesalers
- [ ] Accessing prescription from GP
- [ ] Poor communication regarding delivery dates/time
- [ ] Poor communication between GP and home delivery company
- [ ] Delayed deliveries
- [ ] Missed deliveries
- [ ] Missing items in delivery
- [ ] Wrong items in delivery
- [ ] Poor communication with dietitians when products not available
- [ ] Inedible fresh products due to delay in delivery
- [ ] Unable to find prescription on NHS spine
- [ ] Damaged packaging
- [ ] Broken or damaged food product
- [ ] Out of date foods
- [ ] Food close to use-by

88. Which home delivery service do your **adult patients** use for their LOW PRESCRIPTION FOODS? (**tick all that apply**) *

- [ ] Vitaflo at Home (Vitaflo and Fate products)
- [ ] Homeward (Nutricia/Loprofin products)
- [ ] Dial a Chemist (Mevalia, Promin, Taranis, Metax products)
- [ ] Not applicable

89. Do you have any comments about the previous question?

90. How often are you informed that a GP has refused to **send an adult patient prescription** for LOW PROTEIN FOODS directly to companies? You may have been informed by the GP practice, parent or home delivery company. *

- At least once a week
- Once every 2 weeks
- Once a month
- Rarely
- Unaware that this is an issue

91. Do you have any comments about the previous question?

92. How often **are you** contacted by **home delivery companies** to ask for your help in obtaining a prescription for SPECIAL LOW PROTEIN FOODS for an **adult patient** from the GP practices? *

- At least once a week
- Once every 2 weeks
- Once a month
- Rarely
- Unaware that this is an issue

93. Do you have any comments about the previous question?

94. Commonly GP prescriptions are electronically uploaded to the NHS spine (digtal platform) and this may lead to the prescription for LOW PROTEIN FOODS being downloaded by the wrong supplier e.g. incorrect home delivery company or to a local pharmacy instead of a **designated home delivery company**. How often are you aware that this happens with your **adult patients**? *

- ◯ At least once a week
- ◯ Once every 2 weeks
- ◯ Once a month
- ◯ Rarely
- ◯ Unaware that this is an issue

95. Do you have any comments about the previous question?

96. Does your hospital supply LOW PROTEIN FOOD samples to **adult patients** if they run out? *

- ◯ At least once a week
- ◯ Once every 2 weeks
- ◯ Once a month
- ◯ Rarely
- ◯ Unaware that this is an issue

97. Do you have any comments about the previous question?

98. What are the most common issues related to **adult patients** that affect the supply of LOW PROTEIN PRESCRIPTION FOODS? (**tick all that apply**) *

- ☐ Do not order low protein foods in a timely way
- ☐ Do not collect low protein foods in a timely way
- ☐ Don’t respond to messages from delivery company/chemist
- ☐ Stockpile low protein foods
- ☐ Do not understand ordering process
- ☐ Cannot afford low protein food prescriptions
- ☐ Other

99. Do you have any comments about the previous question?

100. Do **GP practices/local prescribers** place any restrictions on the prescription of LOW PROTEIN FOODS for your **adult patients**? *

- ○ Yes
- ○ No

101. If yes, please describe those restrictions. *

102. What would help in the future to make things better **adult patients** in accessing LOW PROTEIN PRESCRIPTION FOODS? (**tick all that apply**) *

- ☐ Apps specifically produced for ordering special low protein foods
- ☐ Dietitians prescribing
- ☐ Centralisation of system i.e. one supplier
- ☐ Credit card/voucher system for ordering
- ☐ Improved education of GP surgeries/ practice pharmacist
- ☐ Improved liaison with GP surgery/ pharmacist by dietitian/department
- ☐ Improved education of patient’s local pharmacy
- ☐ Other

103. Do you have any comments about the previous question?

104. What is your perception of the impact of prescription issues on your **adult patients** blood phenylalanine control? *

- ○ Never impacts
- ○ Rarely impacts
- ○ Sometime impacts
- ○ Frequently impacts

105. Do you have any comments about the previous question?

106. Do you have any final comments?

This content is neither created nor endorsed by Microsoft. The data you submit will be sent to the form owner.

Microsoft Forms
