# Supplementary material for: UK Patient Access to Low-Protein Prescription Foods in Phenylketonuria (PKU): An Uneasy Path
Source: Nutrients. 2025 Jan 22;17(3):392. doi: 10.3390/nu17030392 (PMC11820046; doi:10.3390/nu17030392)
Supplement: Supplementary file 1 [file nutrients-17-00392-s001.zip › Table S1.pdf]

Table S1. Demographics of respondents

**REGION OF UK**<br>Hospital	**Number of respondents**	**Paediatric (P)/ Adult (A)/ Both (B)**	**Dietitian (D)/ Dietetic Support Worker* (DSW)**	**Number of patients (Paediatric/Adult)**
**England – East**	**4**	**1P, 2A, 1B**	**3D, 1DSW**	
Addenbrookes, Cambridge	3	2A, 1B	2D, 1DSW	10-20/21-50
Norfolk & Norwich	1	1P	1D	10-20
**England – London**	**15**	**7P, 7A, 1B**	**13D, 2DSW**	
Evelina London	2	2P	2D	>150
Guys & St Thomas	5	4A, 1B	4D, 1DSW	>150
Great Ormond Street	5	5P	4D, 1DSW	>150
University College London	3	3A	3D	>150
**England – Midlands**	**16**	**11P, 5A**	**10D, 6DSW**	
Birmingham Children's	4	4P	3D, 1DSW	101-150
University Hospital Birmingham	5	5A	3D, 2DSW	>150
Leicester Royal Infirmary	4	4P	3D, 1DSW	10-20
Nottingham	3	3P	1D, 2DSW	21-50
**England – North East**	**2**	**2B**	**1D, 1DSW**	
Newcastle Upon Tyne	2	2B	1D, 1DSW	51-100/101-150
**England – North West**	**7**	**5P, 2A**	**6D, 1DSW**	
Alder Hey, Liverpool	2	2P	2D	51-100
Manchester Children's	3	3P	2D, 1DSW	101-150
Salford Royal	2	2A	2D	>150
**England – South West**	**4**	**2P, 2A**	**3D, 1DSW**	
Bristol Children's	2	**2P**	1D, 1DSW	51-100
Bristol Royal Infirmary	1	1A	1D	21-50
Southmead, North Bristol	1	1A	1D	51-100
**England – Yorkshire & Humber**	**8**	**5P, 3A**	**5D, 3DSW**	
St Lukes, Bradford	2	2P	1D, 1DSW	51-100
Sheffield Children's	3	3P	2D, 1DSW	51-100
Northern General, Sheffield	3	3A	2D, 1DSW	101-150
**Northern Ireland**	**7**	**3P, 4A**	**7D**	
Belfast Children's	3	3P	3D	101-150
Royal Victoria, Belfast	4	4A	4D	>150
**Scotland**	**4**	**4B**	**2D, 2DSW**	
Scottish IMD Service	4	4B	2D, 2DSW	>150/>150
**Wales**	**4**	**1P, 2A, 1B**	**3D, 1DSW**	
University Hospital, Cardiff	4	1P, 2A, 1B	3D, 1DSW	21-50/51-100
**TOTAL**	**71**	**35 (49%) paediatric<br>27 (38%) adult<br>9 (13%) both**	**53 (75%) dietitians<br>18 (25%) DSWs/ administrators**	

* Includes 1 dietetic administrator
